# Translating medical screening and brief intervention into behavioral health-care practice in work-related settings

**DOI:** 10.1186/1940-0640-7-S1-A61

**Published:** 2012-10-09

**Authors:** Tracy McPherson, Eric Goplerud

**Affiliations:** 1 Department of Substance Abuse, Mental Health and Criminal Justice Studies, National Opinion Research Center, University of Chicago, Chicago, IL, USA

## 

Substantial empirical support exists for alcohol screening and brief intervention (SBI) in medical settings but not in nonmedical settings such as the workplace—a potential but underutilized venue for alcohol SBI. This research aims to translate medical SBI into behavioral health-care practice in US work settings, where millions of workers might be reached annually. The primary objectives of this pilot study are to assess the feasibility of adapting medical SBI practices to telephonic employee assistance programs (EAPs); to develop practical training, implementation, and quality/fidelity monitoring protocols and processes that can be integrated into existing practices; to assess the impact of implementing systematic routine alcohol SBI on key performance measures (rates of screening, alcohol problem identification, treatment initiation); and to assess preliminary outcomes (self-reported alcohol use, mental well-being, and productivity). The multi-site study is being conducted by US EAP providers using pre-test/post-test single-group pre-experimental designs. Screening and brief intervention processes were adapted from the World Health Organization’s alcohol SBI protocol and included systematic screening using the Alcohol Use Disorders Identification Test (AUDIT) during clinical intake, BI using motivational interviewing techniques, referral to face-to-face counseling, and telephonic follow-up. Preliminary findings suggest that integration of routine SBI by EAP consultants at intake is not only feasible in a telephonic delivery system but also increases alcohol problem identification to levels found in the general US population; hence, it provides the opportunity for brief motivational counseling for risky drinking. Furthermore, it is clear that when SBI is integrated as part of routine EAP practice, members are willing to answer questions about their alcohol use and participate in follow-up. To date, findings have shown increases in alcohol-problem identification from 400% to 600% when SBI is integrated into routine practice.

